# Prliminary Medical Education — Regulations of the Buffalo Medical Association

**Published:** 1848-06

**Authors:** 


*Prliminary Medical Education — Regulations of the Buffalo Medical Association.*

In conformity with the recommendation of the American Medical Convention, the Buffalo Medical Association, some time since, adopted regulations, with a view to secure proper preliminary qualifications of students received into the offices of its members. The following is the report of the committee appointed to draft additions to the by-laws of the Association on this subject, which was duly adopted. We publish it in order that the members of the Association may bear the regulations in mind, so as to be governed accordingly; and also in the hope that other associations may adopt similar provisions.

*Report upon the Requirements of Students.*—The Commitee appointed at the last meeting of the Association, to "suggest a plan and rules to regulate the members in the admission of students to their offices," respectfully report.

That in order to uniformity of system, it appears to us proper that the State and County Medical Societies should recommend and adopt rules to govern themselves, in the admission of Students to the study of medicine; but in the absence of any such rules or recommendations, we deem the matter of sufficient present importance to demand of us some *temporary* plan for our own guidance. We therefore recommend to the Association the adoption of the following addition to the By-Laws.

§ 13. To be admitted to the offices of any of the members of this Association, as a student of medicine, it shall be required that the applicant shall, in addition to an English Academic education, have made respectable attainments in the Latin language; and that he shall sustain a good moral character. In evidence of which the applicant shall present the certificate provided for by the *fifteenth section* of these by-laws.

§ 14. A Board of Examiners, consisting of three, to be called the "Primary Board," shall be chosen at the Annual Election, by ballot, whose duty it shall be, whenenever notified, in writing, by any member, to meet and examine the applicant.

§ 15. If the examination is satisfactory, the applicant shall be furnished with a certificate of the fact, in the following form:

We certify that we have examined A——— B———, of C———, and that he has a good English Academic education. We certify also, that he has produced before us a certificate (or certificates, or an affidavit, as the case may be. An affidavit made before a proper officer, may be secured where, for reasons which may seem satisfactory to the Board, a certificate cannot be obtained,) that he has diligently studied the Latin language during six months, (or, that he is a graduate of a literary college.)
We further certify that he has produced before us, a certificate of good moral character.
Signed by at least two members of the Board.

A——— }
B——— } *Primary Board*
[Dated] C——— }

§ 16. The Board shall, at each secular meeting, furnish to the Association of names of those to whom they have given certificates since the last regular meeting, and the Secretary of the Association shall make a faithful record of the same.

§ 17. The first election of the "Primary Board" shall occur whenever these additional by-laws shall be adopted, and the persons then chosen shall hold their office until the next annual meeting.

All of which is respectfully submitted.

JOSIAH TROWBRIDGE, }

W. TREAT, } *Committee.*

F. H. HAMLTON. }

